# An Essay on Heat

**Published:** 1783

**Authors:** 


THIS intereſting paper is the reſult of experiments on heat made at Paris in the courſe of the laſt winter. It conſiſts of 53 pages, printed printed as part of a volume of the Memoirs of the Academy of Sciences at Paris, not yet publiſhed. In this detached form it was given by the authors, to a gentleman who has favoured us with a peruſal of it.

Profeſſor Wilcke, in the Stockholm Memoirs for 1781, mentions an idea he had conceived of melting ſnow by different ſubſtances in order to aſcertain their ſpecific heat. But the difficulty of collecting the water produced by the melting of the ſnow, the length of time bodies require to give out their heat in this way, and the heat which the ſnow acquires from the atmoſphere, obliged him to give up this method, which was defective in this, that the ſnow thus employed was not ſecured from the action of the atmoſphere by an outer covering of ice or ſnow. This defect is very ingeniouſly ſupplied by the authors of the paper before us, by means of a machine, the deſcription of which is illuſtrated by two copper-plates.

This machine conſiſts of three cavities one within the other. The innermoſt is formed of a net work of iron wire, to which a cover of a ſimilar ſtructure is adapted. This receptacle, which is intended for the ſubſtance whoſe heat is to be meaſured, is incloſed by a double caſe of of tin, forming two other cavities, with diſtinct covers, that are filled with ice. The ice which immediately ſurrounds the net work filters, as it melts, into a reſervoir at the bottom of the machine, while the ice in the outer caſe runs off through a diſtinct channel. The whole machine is ſo conſtructed that there cannot be the leaſt communication between the outer layer of ice and that which is in contact with the iron wire, and of courſe the melting of the inner layer will be in proportion only to the heat it receives from the ſubſtance placed in the innermoſt cavity of the machine. The way then in which they determine the ſpecific heat of any ſubſtance, is firſt to heat it to any certain number of degrees above 32° of Fahrenheit's thermometer, and then to place it in the machine in the manner above deſcribed till its temperature is reduced to 32°. The water produced by its cooling is then to be meaſured. This quantity of water divided by the product of the maſs of the body, and of the number of degrees of its original temperature above 32°, will be in proportion to its ſpecific heat.

This method, we are told, is applicable to all the phænomena in which there is extrication or abſorption of heat, and will enable us to aſ-

certain certain and compare with each other the quantity of heat produced by mixing oil of vitriol with water, water with quick lime, quick lime with nitrous acid, &c. or that which is dissipated in the combuſtion of phoſphorus or of ſulphur, in the detonation of nitre, in animal reſpiration, &c. all which was impracticable by the methods hitherto known.

In the ſecond of the four ſections into which the Eſſay is divided, the authors give the reſult of the principal experiments made according to this method. In the third they examine the concluſions which may be drawn from thoſe experiments, and in the fourth they treat of combuſtion and reſpiration. Theſe parts of the Eſſay do not allow of abridgment.

